# Design and Pharmacological Characterization of a Novel Antithrombotic P2Y_1_ Receptor-Based Vaccine

**DOI:** 10.3390/ijms26094383

**Published:** 2025-05-05

**Authors:** Osaid Al Meanazel, Fatima Z. Alshbool, Fadi T. Khasawneh

**Affiliations:** 1 Department of Pharmacy Practice, College of Pharmacy, Texas A&M University, Kingsville, TX 78363, USA; otalmeanazel@tamu.edu (O.A.M.); falshbool@tamu.edu (F.Z.A.); 2 Department of Pharmaceutical Sciences, College of Pharmacy, Texas A&M University, Kingsville, TX 78363, USA

**Keywords:** platelets, thrombosis, aggregation, vaccine

## Abstract

Platelet activation processes begin when injury to blood vessels exposes the subendothelial matrix, leading platelets to attach to it, where they become activated and exert their hemostatic function. Excessive platelet aggregation is associated with thrombotic disorders such as arterial thrombosis. To manage such diseases, medications that inhibit thrombosis are continuously sought, despite potential drawbacks that include hemorrhage. This study described the development of a novel peptide-based vaccine that targets the purinergic ADP P2Y_1_ receptor (abbreviated EL2Vac) and its pharmacological characterization. Thus, we designed and developed an EL2Vac that targets the ligand-binding domain of the P2Y_1_ receptor protein, which is located in its second extracellular loop (EL2). We then evaluated the vaccine’s ability to trigger an immune response (antibody production) in immunized mice, modulate platelet function, its antithrombotic activity, and any effects on hemostasis, by employing a thrombosis model and the tail bleeding time assay. Results showed significant levels of antibody production in mice treated with EL2Vac, in comparison with the random peptide vaccine control (EL2rVac), which persisted at least up to six months post vaccination. Moreover, we observed significant inhibition of the ADP-induced aggregation response in platelets from EL2Vac-treated mice, relative to those from EL2rVac controls. This inhibition was selective for ADP, as other agonists, such as the thromboxane A_2_ receptor (TPR) agonist U46619 or high-dose collagen, had no detectable effect on aggregation. As for its capacity to protect against thrombosis, our data showed a significant delay in the occlusion time of the EL2Vac mice when compared with the random peptide control vaccine, which was also observed (at least) six months post vaccination. Interestingly, EL2Vac did not appear to prolong the tail bleeding time, supporting the notion that it is devoid of a bleeding diathesis. As a conclusion, this study documents the design and evaluation of a novel peptide-based vaccine, EL2Vac, which appears to selectively target the P2Y_1_ receptor and protect against thrombus formation without impairing hemostasis. Thus, EL2Vac may provide a promising clinical option to treat thromboembolic disorders.

## 1. Introduction

Hemostasis is the process of maintaining the blood vessels’ integrity after vascular injury by forming a clot at the site of the wound. This can be attained, in part, through interactions involving endothelial cells, platelets, and the coagulation cascade. Platelets are anucleate discoid-shaped cells that do not interact with the vessel wall unless the subendothelial matrix gets exposed, allowing them to attach and get activated through surface receptors and adhesion molecules. Thus, the primary mechanism for hemostasis is initiated through platelet adhesion to the uncovered subendothelial matrix upon injury, subsequent activation, and aggregation. Furthermore, platelets can act as a promoter for the coagulation cascade through their procoagulant surface, which is known as secondary hemostasis [1,2]. One important platelet surface receptor family is the G-protein-coupled receptors (GPCRs), which are essential for platelet activation and aggregation [1,3,4]. Among the various platelet activators, adenosine 5′-diphosphate (ADP) is secreted from the dense granules and is responsible for their activation via interaction and subsequent stimulation of two separate GPCRs: the purinergic P2Y_1_ and P2Y_12_ receptors (P2Y_1_-R and P2Y_12_-R) [5,6,7].

The P2Y_1_-R and P2Y_12_-R receptors are responsible for the “total” platelet aggregation triggered by ADP. Activation of the P2Y_12_-R receptor leads to intensification and maintenance of the aggregation response by inhibiting the Gi-dependent adenylyl cyclase activity and decreasing the cyclic adenosine monophosphate (cAMP) levels. Moreover, P2Y_1_-R activation is thought to be essential for platelet shape alteration and also plays a pivotal role in ADP-induced aggregation through Gq-dependent phospholipase C (PLC) stimulation that results in the production of inositol triphosphate (IP3) and, consequently, the release of intracellular calcium [8,9]. Regardless of its vital role in hemostasis, excessive aggregation can lead to serious conditions that include arterial thrombosis [10,11]. Consequently, P2Y_12_ antagonists (e.g., clopidogrel (plavix^®®^)) have been extensively used to prevent the propagation of platelet aggregation due to their prominent role in this functional response in comparison to P2Y_1_, but such a strategy also showed a high risk of bleeding [12,13,14,15,16,17]. It is noteworthy that, thus far, there are no P2Y_1_-R antagonists that have been approved for clinical use. However, Fabre et al. demonstrated a vital role of P2Y_1_-R in platelet aggregation, using P2Y_1_-R deficient mice that were found to exhibit impaired ADP-induced platelet aggregation and delayed thrombosis [13], which were supported by a separate report by Lenain et al. [18]. To this end, the antagonism of the P2Y_1_ receptor has garnered some interest in light of the transgenic and pharmacological approaches that clearly document it as a mediator for thrombotic disease (shown in Table 1) [13,18,19,20]. Nevertheless, those antagonists have not been clinically approved, due to various limitations, and as far as we know (http://clinicaltrials.gov/), there are no ongoing clinical trials evaluating any P2Y_1_-R antagonist [21]. It is noteworthy that the P2Y_1_-R antagonists had generally been designed based on the structure of ADP and ATP, regardless of the P2Y_1_-R ligand-binding domain.

In our pursuit of P2Y_1_-R inhibitors, we developed an antibody targeting its ligand-binding domain, namely the second extracellular loop (EL2) of the receptor protein [31]. Our results demonstrated that this antibody, abbreviated EL2Ab, effectively and selectively inhibited ADP-induced platelet function and protected against thrombogenesis.

**Figure 1:**
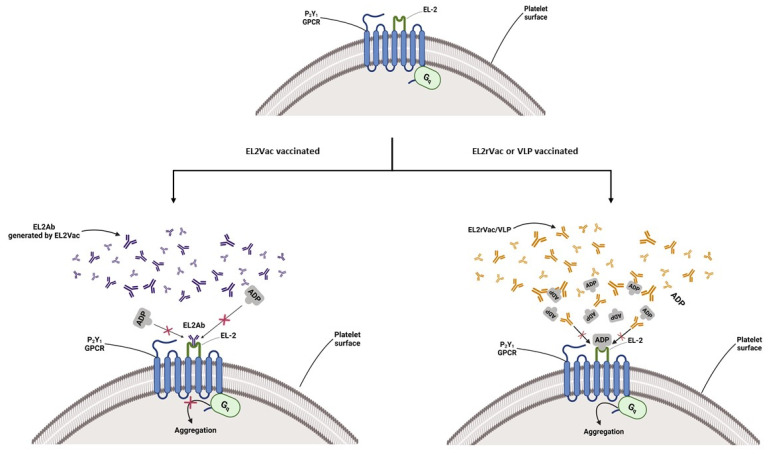
A cartoon model depicting potential inhibition of ADP-induced activation of the P2Y_1_-R by EL2Vac. Vaccination with EL2Vac will trigger an immune response and generate antibodies targeted against the EL2 of P2Y_1_-R, which will prevent ADP from binding, thereby inhibiting ADP-induced platelet activation.

In this context, vaccines have long been established as a therapeutic approach for a host of diseases/infections, and more recently, for managing HIV, HPV, and cancers [32,33,34]. This is especially the case for peptide-based vaccines due to their proven safety profile, low cost, ease of manufacturing, and, most importantly, better patient compliance [35]. In this context, peptide vaccines use a 20–30 amino acid sequence to produce an immunogenic peptide representing a particular epitope, which would be sufficient to develop an appropriate cellular and humoral response [36,37]. Based on the aforementioned considerations, the present study aims to design and characterize a peptide-based vaccine modeled on the EL2 domain of P2Y_1_-R (EL2Vac), relative to the random EL2 peptide vaccine control (EL2rVac), for its antithrombotic activity (Figure 1).

## 2. Results

### 2.1. Quantification of Antibody Production in Vaccinated Mice

**Figure 2:**
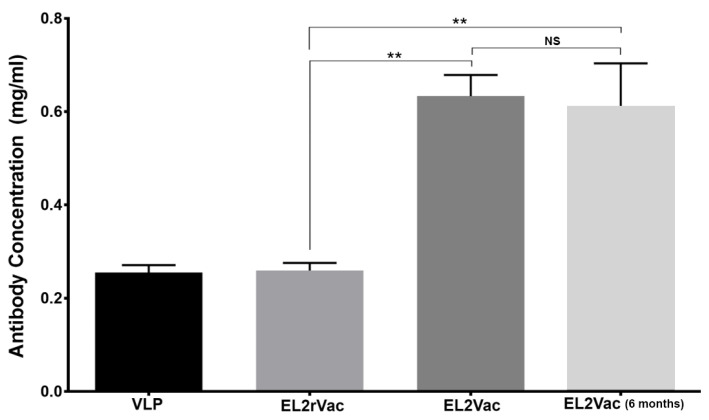
Antibody concentration in mice vaccinated with EL2Vac, EL2rVac, or VLP. Mice were immunized with VLP coupled with the EL2 sequence (CGGTGVRKNK-TITCYDTTSDEYLRSYF; T192-F215), random EL2 (LYKDGTICTYVFYNKRRSDTETST), or VLP. EL2Ab, which was measured, including after six months, in the vaccinated mice using ELISA and the EL2 cognate peptide, was detectable at significant levels in the EL2Vac mice, but not the EL2rVac and VLP controls. The titers were assessed in five distinct vaccination groups, each comprising five mice (** *p* ≤ 0.001); the abbreviation NS denotes insignificance (*p* ≥ 0.05).

To confirm that vaccination with EL2Vac efficiently induce an immune response; serum samples were collected from mice vaccinated with the EL2 peptide (EL2Vac), or the random EL2 peptide (EL2rVac), and virus like particles (VLP) controls, and the concentration of the antibody produced was determined using ELISA utilizing the EL2 cognate peptide. The EL2Vac was found to result in the production of significant levels of antibody, including up to six months post vaccination, unlike EL2rVac and VLP (Figure 2). Thus, this result indicates that the EL2Vac approach was successful in generating antibodies against EL2 (EL2Ab) that would presumably interact with the EL2 region of P2Y_1_-R and exert a biological effect.

### 2.2. Platelet Counts in Vaccinated Mice

Since previous studies showed an altered platelet count due to vaccine-induced immunoresponses [38,39,40], we sought to determine whether EL2Vac would produce any effects. The results indicated no significant difference in the platelet counts between the EL2Vac mice, in comparison with the EL2rVac and VLP groups, suggesting that EL2Ab production and interaction with the P2Y_1_-R do not affect the platelet life span (Table 2).

### 2.3. EL2Vac Inhibits ADP-Induced Platelet Aggregation

**Figure 3:**
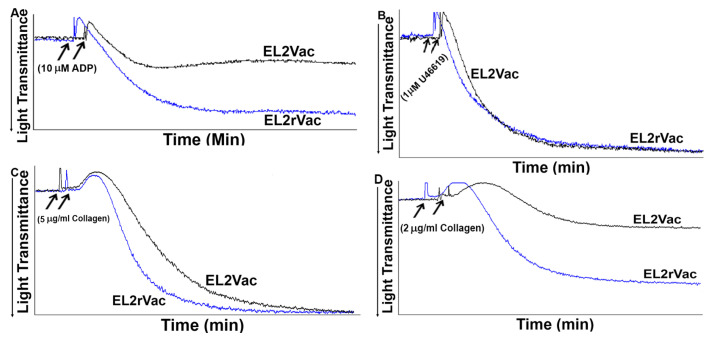
Platelet aggregation assessment in the vaccinated mice. Platelets were obtained from mice vaccinated with 20 µg of EL2Vac or EL2rVac and stimulated with three different agonists, (**A**) 10 µM ADP, (**B**) 1 µM U46619, (**C**) 5 μg/mL collagen or (**D**) 2 μg/mL collagen before their aggregation response was measured in a lumi-aggregometry system. EL2Vac showed selective inhibition of ADP- and low-dose collagen-induced platelet aggregation. Each test was performed in triplicate using three separate groups, with blood pooled from six mice per group each time.

Our previous work had demonstrated the ability of a custom-made antibody against the EL2 domain of P2Y_1_-R to block ADP-induced platelet aggregation [31]. In the current study, we assessed whether EL2Vac would exhibit a similar activity. Indeed, EL2Vac was found to exert significant inhibitory effects on platelet aggregation induced by 10 µM ADP, whereas EL2rVac had no effect (Figure 3A). To evaluate its specificity, the capacity of EL2Vac to inhibit non-ADP-mediated platelet aggregation was examined. As one might expect, EL2Vac had no apparent effect on platelet aggregation induced by 1 µM of the TPR agonist, U46619, or a high dose (5 μg/mL) of collagen (Figure 3B,C). Of note, low-dose collagen-triggered platelet activation is thought to involve P2Y_1_-R [19]. Interestingly, and consistent with this notion, when a low dose (2 μg/mL) of collagen was utilized, significant inhibition was observed (Figure 3D). Taken together, these data support the selectivity of EL2Vac for P2Y_1_-R.

### 2.4. EL2Vac Inhibits ADP-Induced Alpha Granule Secretion

**Figure 4:**
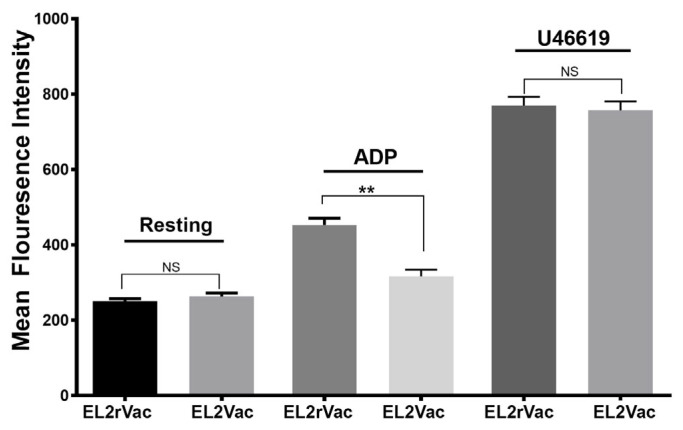
P-selectin expression assessment in the vaccinated mice. Platelets were obtained from mice vaccinated with EL2Vac or EL2rVac and stimulated with two different agonists, 10 µM ADP or 1 µM U46619, before their alpha granule secretion/P-selectin expression was measured using flow cytometry. This experiment was conducted in triplicate, and blood was collected from eight mice and pooled per group. The error bars represent the SEM, whereas NS denotes insignificance (** *p* ≤ 0.001).

Next, we examined P-selectin expression on the surface of activated platelets as a measure of secretion of alpha granules in the context of our antithrombotic vaccine. EL2Vac was found to exert significant inhibition of P-selectin expression following activation by ADP, whereas the EL2rVac control showed no effect (Figure 4). Moreover, neither EL2Vac nor EL2rVac exhibited any activity against U46619 (Figure 4). These results are consistent with our previous work using EL2Ab [31].

### 2.5. EL2Vac Prolongs the Occlusion Time Without Affecting the Tail Bleeding Time

**Figure 5:**
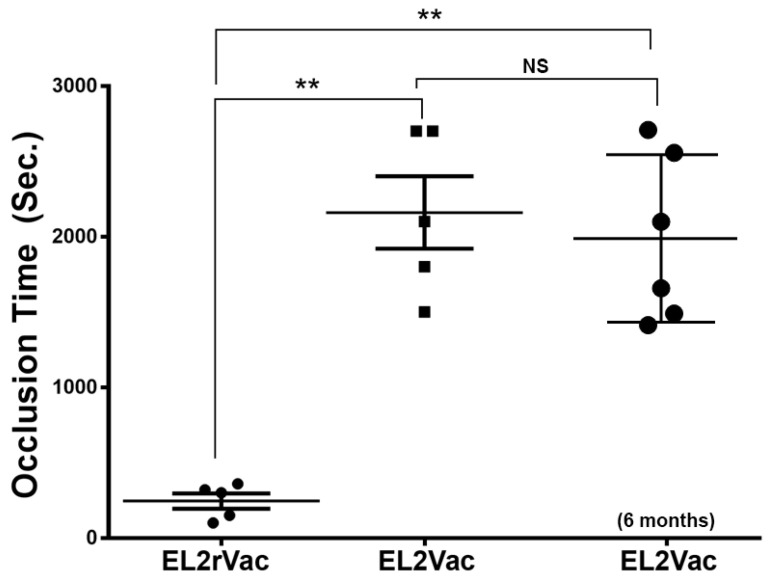
Occlusion time in the vaccinated mice. Mice vaccinated with EL2Vac or EL2rVac were subjected to the FeCl_3_-induced carotid artery injury thrombosis model. The time for occlusion was found to be delayed in EL2Vac, unlike EL2rVac, and it persisted six months later. Each point represents a single animal (*n* = 5 for EL2rVac and EL2Vac, and *n* = 6 for the EL2Vac 6-month experiment); NS denotes insignificance, ** *p* ≤ 0.001.

In the next series of experiments, thrombogenesis was investigated following our vaccinations using the FeCl_3_-induced thrombosis model. Our data, as shown in Figure 5, revealed that EL2Vac, but not EL2rVac, significantly prolonged the time needed for occlusion of the carotid artery; this effect was found to persist at least six months post vaccination (Figure 5). These data show the ability of the developed vaccine to prevent thrombus formation and support its potential clinical use for managing thrombotic disorders.

**Figure 6:**
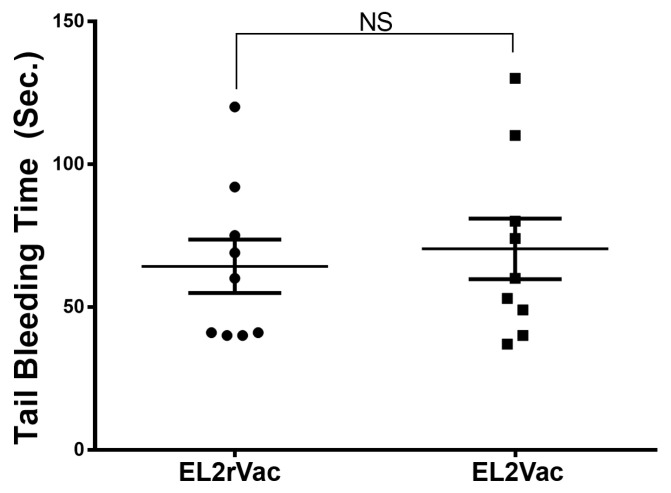
Tail bleeding time in the vaccinated mice. Mice vaccinated with EL2Vac or EL2rVac were subjected to the tail bleeding time assay. The bleeding time was not impacted in the EL2Vac or EL2rVac mice. Each point represents a single animal (*n* = 9 for EL2rVac and EL2Vac). The abbreviation NS denotes insignificance (*p* ≥ 0.05).

We next decided to evaluate the capacity of our vaccination-based approach to modulate hemostasis by using the tail bleeding time assay. Interestingly, EL2Vac did not exert any detectable effects, much like the control EL2rVac (Figure 6). Taken together, these findings support the notion that EL2Vac exerts thromboprotective activity without modulating hemostasis/increasing the risk of bleeding.

### 2.6. EL2Vac Does Not Inhibit the P2Y_12_-R

**Figure 7:**
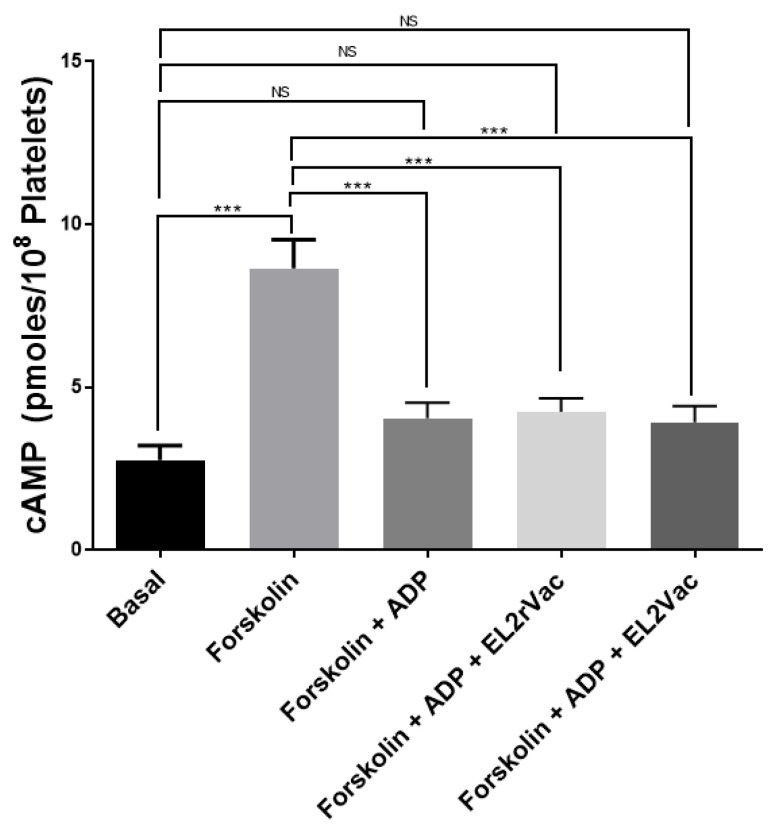
The effects of EL2Vac on ADP-triggered reduction in cAMP levels in platelets. cAMP levels were measured in EL2rVac and EL2Vac mice, as discussed in Methods. The experiment was conducted in triplicate, and blood was collected from five to six mice and pooled per group. *** *p* ≤ 0.0001; the abbreviation NS denotes insignificance (*p* ≥ 0.05).

Our results do not address whether EL2Vac interacts/interferes with P2Y_12_-R-mediated platelet activation. To address this issue, we assessed the capacity of EL2Vac to inhibit ADP-mediated reduction in cAMP in the presence of forskolin, as we previously described [31]. Our results showed that neither EL2rVac nor EL2Vac exerted any effects on the ability of ADP to lower the cAMP levels that are elevated using forskolin [31] (Figure 7).

## 3. Discussion

The primary hemostatic pathway for any sustained injuries in blood vessels involves platelet activation, which will result in the initiation of subsequent events, including aggregation and secretion [41,42,43]. Thus, injury-induced exposure of the subendothelial tissues enables platelets to adhere and then promote the formation of a plug to prevent further hemorrhage. At the same time, attachment of platelets to the exposed subendothelial matrix will trigger a series of intraplatelet signaling and activation cascades, including release of ADP from the granules [44], which will subsequently interact with its GPCRs on the platelet surface, specifically, P2Y_1_-R and P2Y_12_-R [3,4,6,7]. ADP-mediated activation will also initiate the conformational changes of the GP IIb-IIIa to facilitate fibrinogen binding and, thus, aggregation of the platelets [45,46]. Despite the significant role of platelet aggregation in maintaining hemostasis of blood vessels, their unregulated and/or hyperactivation is one of the causes of arterial thrombosis.

With regard to the ADP GPCRs, signaling downstream of P2Y_12_-R has been found to play a more prominent role in platelet activation than that of the P2Y_1_-R [47,48]; however, that appears to come at the expense of a significant underlying bleeding phenotype upon pharmacological targeting/antagonizing the P2Y_12_-R. Consequently, antagonism of the P2Y_1_-R is thought to lead to a less potent and perhaps safer alternative. Thus far, due to the limitations of the designed P2Y_1_-R antagonists, none have yet received approval by the FDA [21]. This is attributed to the fact/drawback that their design is based on the structure of ADP and/or ATP, neglecting the ligand-binding domain on the P2Y_1_-R protein. To this end, based on research indicating that the P2Y_1_-R ligand-binding domain for ADP is located on the EL2 region of the receptor protein, we developed and showed that an antibody against this region (EL2Ab; passive immunization) is able to selectively inhibit ADP-induced platelet function (it did not appear to exert effects on P2Y_12_-R), and protect from thrombogenesis, without adverse bleeding [31]. In light of the limitations of antibody-based therapies, we sought to develop a peptide-based vaccine modeled over EL2. This vaccine is expected to initiate an immune response (active immunization) in the host and produce “EL2Ab” while maintaining the same pharmacological activity and safety profile. Indeed, ELISA-based antibody quantification revealed robust levels of EL2Ab in mice injected with EL2Vac, whereas it was undetectable in the control mice (EL2rVac or VLP). This indicates that the EL2 peptide-based vaccine elicits an immune response and generates an antibody against the EL2 domain, unlike the random peptide vaccine or the VLP. Moreover, the immune response appears to last for at least six months after the last booster, suggesting somewhat of a prolonged response, which would be helpful for compliance purposes, and for reducing the need for “frequent dosing”. Thus, individuals who are at high risk of developing thrombotic events can benefit from this vaccine in terms of prophylaxis. Moreover, it is also possible that it can be used for secondary prevention in those who have had a previous event.

In a separate set of studies, we investigated the pharmacological activity of EL2Vac and its safety in the context of bleeding. The results showed selective inhibition of ADP-induced platelet aggregation by EL2Vac, whereas EL2rVac had no effect. This suggests that the vaccine-generated EL2Ab interacts with the ADP binding site and blocks the P2Y_1_-R. This is also consistent with the finding that EL2Vac inhibited low-dose, but not high-dose, collagen-induced platelet aggregation, and the notion that the former involves P2Y_1_-R [19]. Furthermore, based on the connection between P2Y_1_-R and thrombotic disorders [3,13,22,24,25,49], EL2Vac is likely to exhibit antithrombotic activity. Indeed, when the EL2Vac mice were subjected to a widely used thrombosis model, the time of occlusion was significantly delayed in contrast to those vaccinated with EL2rVac. Moreover, these protective effects against thrombogenesis were observed six months post vaccination, supporting a somewhat “long duration” of activity. These data provide further evidence of the integral role P2Y_1_-R has in the genesis of thrombosis, and that a vaccine based on its ligand-binding domain could exhibit thrombo-protective effects. Interestingly, and with regard to its safety, the EL2Vac had no apparent effect on the bleeding time, which indicates that normal hemostasis can be maintained; and is also consistent with the notion that P2Y_1_-R may present a “safer” therapeutic target. It is important to note that findings with EL2Vac appear to be “similar” to those observed with our TPR vaccine [50], as well as the serotonin 5HT_2A_R vaccine [51], but differ from those with the custom P2Y_1_-R antibody (EL2Ab) [31], and the small molecule antagonists of 5HT_2A_R, e.g., AR246686 [52] that exhibited effects on bleeding. This likely occurs because the antibody cannot penetrate the endothelial cell layer, and hence will not reach the smooth muscle cells. Thus, this approach seems to have enabled us to address bleeding and thrombosis under conditions of selective modulation of the platelet P2Y_1_-R, but not those of the smooth muscle, which are known to affect bleeding time. Additionally, our data demonstrated that EL2Vac does not inhibit P2Y_12_-R, which is also consistent with the lack of effect on the bleeding time, given the critical role this receptor protein plays in platelet function. Of note, our previous study involving the antibody targeting the EL2 domain of P2Y_1_-R (the passive immunization approach) also indicated a lack of interaction with P2Y_12_-R [31].

Thrombocytopenia, which had been associated with some of the antibody/vaccine-based therapeutic approaches, was also evaluated in the present study. We did not observe any changes in the platelet counts as a result of EL2Vac, which might be expected, as similar results were found with cocaine and angiotension I (Ang I) vaccines [53,54], which further supports the safety of EL2Vac. While we cannot exclude the possibility that such treatments may cause thrombocytopenia at later time points (and in humans), these findings nonetheless suggest that EL2Vac appears to be devoid of thrombocytopenia. Of note, we did not observe any effects on the weight of mice, physical appearance, etc.

Additional characterization of the antiplatelet activity of EL2Vac, namely its effects on alpha granule secretion, revealed that it significantly and selectively reduced P-selectin expression in response to activation by ADP/P2Y_1_-R but not TPR. These findings indicate that, as expected, the EL2Ab generated due to EL2Vac selectively binds to P2Y_1_-R, thereby inhibiting ADP-induced platelet activation.

Collectively, these findings support the notion that EL2Vac has the potential to serve as a novel therapeutic agent without exerting any adverse bleeding effects, albeit these issues will have to be further determined in human subjects.

## 4. Materials and Methods

### 4.1. Materials and Animals

Disposables (e.g., glass cuvettes) related to platelet aggregation, as well as collagen, were purchased from Chrono-Log (Havertown, PA, USA). The thromboxane A_2_ receptor agonist U46619 was obtained from Cayman Chemical (Ann Arbor, MI, USA). ADP, VLP, as well as complete and incomplete Freund’s adjuvants, were purchased from Sigma Aldrich (St. Louis, MO, USA). The anti–P-selectin antibody was obtained from Cell Signaling Technology, Inc. (Danvers, MA, USA). The BD FACSTM lysing solution was purchased from BD Biosciences (Franklin Lakes, NJ, USA).

C57BL/6J mice, mixed gender aged 8–10 weeks, were purchased from Jackson Laboratories (Bar Harbor, ME, USA) and kept under normal conditions (temperature of 24 °C, 12 light cycles) while divided into groups of 1–4 mice at each group with ad libitum access to water and food. Any experiment performed on the animals was approved prior to their use by the Institutional Animal Care and Use Committee.

### 4.2. Methods

#### 4.2.1. EL2 and EL2Ab Peptide Based Vaccination Protocol

Mice were vaccinated using a peptide linked to VLP, corresponding to the human EL2 sequence (i.e., CGGTGVRKNKTITCYDTTSDEYLRSYF; T^192^-F^215^) of the purinergic P2Y_1_-R. Control mice were immunized with a random version of EL2 (i.e., CGGLYKDGTICTYVFYNKRRSDTETST). In each case, an additional sequence of amino acids, specifically CGG,s was synthesized to allow directional conjugation to VLP. Of note, VLP alone was also utilized as a control in the initial experiments to evaluate the immune response triggered by the vaccinations. The vaccines (20 µg) and VLP dissolved in Freund’s complete adjuvant were injected into the mice intraperitoneally. The mice received three additional boosts of peptides (using Freund’s incomplete adjuvant) on days 14, 28, and 42. Unless stated otherwise, the described analyses were conducted two weeks after completing the immunization regimen (day 56).

#### 4.2.2. Quantification of Antibody Production in the Vaccinated Mice

ELISAs were conducted to assess antibody production in the vaccinated mice, namely EL2Vac, EL2rVac, and VLP. Of note, the EL2Vac levels were also assessed six months post the last booster immunization. Nunc-Immuno MicroWell 96-well plates were coated with cognate EL2 peptide at 12.5 µg per well and incubated at room temperature for 18–24 h. Next, the plates were washed (3X) with modified Tyrode’s buffer before being treated with 5% BSA in modified Tyrode’s buffer for 1 h to prevent nonspecific binding. The plates were washed for another time (3X) with modified Tyrode’s buffer, and the EL2 peptide was added to the wells in triplicate using serial dilutions. The antibodies presumably generated by the vaccination (EL2Ab) were incubated for 1 h, washed, and those attached to the immobilized peptide were detected by incubating the wells with goat anti-mouse IgG (heavy and light chains) labeled with horseradish peroxidase for 1 h. The wells were washed one final time before introducing the horseradish peroxidase substrate solution. Following a 10 min incubation in darkness, the reaction was stopped using 2 N H_2_SO_4_, and the antibody’s presence was evaluated by obtaining the absorbance at 490 nm, as previously described [50].

#### 4.2.3. Effect of the Peptide-Based Vaccines on Platelet Aggregation

Blood was obtained from mice that had received the EL2Vac and EL2rVac vaccines. Platelets were obtained through centrifugation. Platelet aggregation was carried out using a Model 700, 2-channel whole blood/optical Lumi-Aggregometry System (Chronolog), at 37 °C with stirring (1000 rpm). The platelet counts were adjusted following Manolopoulos P. et al. and Gearing K. et al. protocols [55,56], before being activated using 10 µM ADP, 1 µM U46619, 2 μg/mL collagen, or 5 μg/mL collagen, and their aggregation response was measured.

#### 4.2.4. Effect of the Peptide-Based Vaccines on Surface Expression of P-Selectin

Flow cytometry experiments were executed on platelets obtained from vaccinated mice (EL2Vac and EL2rVac), as per our previously established protocol [31]. In summary, platelets (at a concentration of 2.0 × 10^8^) were activated with 10 µM ADP or 1 µM U46619 for 3 min, and the reactions were stopped by fixing the platelets with 2% formaldehyde for 30 min at 20–25 °C. Subsequently, the platelets were incubated with anti-P-selectin antibody at room temperature in the dark for 30 min. Finally, platelets were diluted 2.5-fold using HEPES/Tyrode’s buffer (pH 7.4), samples transferred to fluorescence-activated cell sorting tubes, and fluorescence intensities were assessed using a flow cytometer (BD Accuri C6, BD Biosciences, San Jose, CA, USA). The collected data were subjected to analysis using the CFlow Plus software (Version 1.0.34.1).

#### 4.2.5. Effect of the Peptide-Based Vaccines on FeCl_3_-Induced Thrombosis

The flow measurements of the carotid artery FeCl_3_-induced thrombosis in the vaccinated mice (EL2Vac and EL2rVac) were performed using a Perivascular Transit-time Flowmeter (Transonic Systems, Ithaca, NY, USA). In brief, mice that had been vaccinated were anesthetized using isoflurane before the left carotid artery was exposed and cleaned. The initial blood flow in the carotid artery was measured using a Transonic microflow probe (0.5 mm; Transonic Systems Inc., Ithaca, NY, USA). Once the blood flow stabilized, a filter paper disc (diameter of 1 mm) immersed in 7.5% FeCl_3_ was applied to the artery for 3 min, and the blood flow was continually monitored for 45 min or until the blood flow reached a stable occlusion state (with no blood flow for 2 min). The time taken for the vessel to occlude was determined by calculating the time difference between achieving stable occlusion and removing the FeCl_3_ filter paper. For statistical analysis, an occlusion time of 45 min was set as the threshold [57].

#### 4.2.6. Effect of the Peptide-Based Vaccines on the Tail Bleeding

The process of hemostasis in the vaccinated mice (EL2Vac and EL2rVac) was evaluated using the tail transection method, in order to assess bleeding as a potential adverse event. In brief [58], the mice were anesthetized using isoflurane and positioned on a homeothermic blanket at 37 °C. The tails of the vaccinated mice were then transected at a point 5 mm away from the tip, and immediately immersed in 37 °C saline solution. The duration until stoppage of blood flow/bleeding was recorded. If needed, measurements were concluded at the 10 min mark to prevent excessive blood loss.

#### 4.2.7. Effect of the Peptide-Based Vaccine on cAMP Levels

The cAMP assay was conducted as we described before [31]. Mouse PRP (500 µL) samples were collected from the EL2rVac and EL2Vac mice. Next, platelets are treated with 0.5 µM forskolin before the addition of 10 µM ADP, and incubated at room temperature for 1 min. Next, the phosphodiesterase inhibitor RO20-1724 (100 µM) was added, and platelets were spun down, and the pellet was snap frozen in liquid nitrogen and stored at −70 °C. Upon use, the pellet is resuspended in sodium acetate buffer (50 mM; pH 4.0), sonicated, boiled for 4 min, centrifuged, and the supernatant transferred to a new tube. The concentration of cAMP in the supernatant is measured according to standard protocols [59], whereas the standard curve samples were prepared by adding known concentrations of cAMP to the supernatant from the EL2rVac platelets.

#### 4.2.8. Statistical Analysis

Every experiment was repeated a minimum of three times, except the in vivo ones that were conducted on 5–9 animals as applicable. Data analysis was conducted using GraphPad PRISM (Version 7.0) statistical software (San Diego, CA, USA). For assessing differences in mean occlusion and bleeding times, the Mann–Whitney test was employed. The flow cytometry and ELISA data were analyzed using a Student’s *t*-test. The data are expressed as the mean ± SEM. Results were considered statistically significant when the *p*-value was less than 0.05 (using a 2-tailed *p* value) unless otherwise specified.

## 5. Conclusions

In summary, our study describes the design and characterization of a novel approach to managing thrombotic disorders, which involves a peptide-based immunization approach/vaccine modeled on the ligand-binding site (EL2) of the platelet P2Y_1_-R. EL2Vac exhibits antiplatelet and antithrombotic effects, without causing adverse bleeding. This study provides further evidence regarding the ADP-ligand-binding site and its importance in regulating platelet function and thrombogenesis. Further studies are needed to explore the potential therapeutic utility of such a peptide-based vaccine in the treatment of thrombotic disorders in humans.

## 6. Patents

Texas A&M University filed a provisional patent for the vaccine reported in the present manuscript with the US Patent and Trademark Office.

## Figures and Tables

**Table 1:** P2Y_1_-R antagonists: pharmacological action and limitations.

P2Y_1_-R Antagonist	Pharmacological Action	Limitations	Reference
A2P5P and A3P5P	Complete inhibition of platelet aggregation	Not selective	[22,23]
MRS2179	Inhibition of platelet function and delay in thrombosis formation	Transient action requiring high doses	[4,6,18,24,25]
MRS2500	Enhanced inhibition of ADP-induced aggregation, antithrombotic activity, higher targeting ability than MRS2179	Potential for irreversible inhibition when used with clopidogrel	[26,27,28]
Di-aryl urea	Inhibited ADP/P2Y_1_ receptor-mediated platelet aggregation in vitro	Unknown in vivo effectiveness, short biological half-life, toxicity	[29,30]
Benzofuran-substituted urea derivatives	Inhibited P-selectin expression	Poor potency, oral bioavailability issues	[29,30]

**Table 2:** Platelet counts for the reference (Basal), Vehicle, EL2Vac, and VLP. All counts were determined individually for each mouse (Five mice per group) and in triplicate, while data in the table represent the mean ± SEM for each group.

Treatment	Mean Platelet Count ± SEM (X × 10 ^−3^/µL)
Basal	825 ± 87
Vehicle (5 days)	829 ±94
Vehicle (14 days)	818 ± 75
EL2Vac 20 µg (≈8 weeks)	841 ± 72
VLP (≈8 weeks)	835 ± 86

## Data Availability

We are not able to make the data available to other researchers for purposes of reproducing the results or replicating the procedure at this time, because of patent considerations. However, once the US Patent and Trademark Office makes a final decision on the patent application pertaining to the present “vaccine,” requests will be honored on a case-by-case basis and will be provided by the corresponding author.
